# Nutritional Support of Chronic Obstructive Pulmonary Disease

**DOI:** 10.3390/nu17071149

**Published:** 2025-03-26

**Authors:** Péter Simon, Éva Török, Klára Szalontai, Beáta Kari, Patrícia Neuperger, Norma Zavala, Iván Kanizsai, László G. Puskás, Szilvia Török, Gabor J. Szebeni

**Affiliations:** 1 National Korányi Institute of Pulmonology, 1121 Budapest, Hungary; szajmon1@gmail.com; 2 Gastroenterology Center Buda, 1117 Budapest, Hungary; torokeva.diet@gmail.com; 3 Department of Pulmonology, Szent-Györgyi Albert Medical Center, University of Szeged, 6772 Deszk, Hungary; hneszalontai@gmail.com; 4 Laboratory of Functional Genomics, Core Facility, HUN-REN Biological Research Centre, 6726 Szeged, Hungary; kari.beata@brc.hu (B.K.); neuperger.patricia@brc.hu (P.N.); normazavpre@gmail.com (N.Z.); laszlo@avidinbiotech.com (L.G.P.); 5 Avidin Ltd., 6726 Szeged, Hungary; 6 Anthelos Ltd., 6726 Szeged, Hungary; 7 Department of Internal Medicine, Hematology Centre, Faculty of Medicine, University of Szeged, 6725 Szeged, Hungary

**Keywords:** COPD, nutrition, vitamins, inorganic elements

## Abstract

**Background**: COPD is a heterogenous disease of the respiratory tract caused by diverse genetic factors along with environmental and lifestyle-related effects such as industrial dust inhalation and, most frequently, cigarette smoking. These factors lead to airflow obstruction and chronic respiratory symptoms. Additionally, the increased risk of infections exacerbates airway inflammation in COPD patients. As a consequence of the complex pathomechanisms and difficulty in treatment, COPD is among the leading causes of mortality both in the western countries and in the developing world. **Results**: The management of COPD is still a challenge for the clinicians; however, alternative interventions such as smoking cessation and lifestyle changes from a sedentary life to moderate physical activity with special attention to the diet may ameliorate patients’ health. Here, we reviewed the effects of different dietary components and supplements on the conditions of COPD. **Conclusions**: COPD patients are continuously exposed to heavy metals, which are commonly present in cigarette smoke and polluted air. Meanwhile, they often experience significant nutrient deficiencies, which affect the detoxification of these toxic metals. This in turn can further disrupt nutritional balance by interfering with the absorption, metabolism, and utilization of essential micronutrients. Therefore, awareness and deliberate efforts should be made to check levels of micronutrients, with special attention to ensuring adequate levels of antioxidants, vitamin D, vitamin K2, magnesium, and iron, as these may be particularly important in reducing the risk of COPD development and limiting disease severity.

## 1. Background

Poor nutritional status, unintentional weight loss, or shifts in body composition are frequent and relevant issues in COPD patients [1,2]. However, the link between nutritional abnormalities and COPD is complex and bidirectional, as nutritional status can serve as both a contributing factor to the onset or the severity of the disease and a consequence of the advanced respiratory condition, thus creating a vicious circle [3,4,5]. Additionally, nutritional imbalance also correlates with susceptibility to infections that can lead to more severe disease and trigger exacerbations. Furthermore, it can result in different comorbid conditions that also increase the all-cause mortality of COPD patients [6,7,8]. Dietary guidance and oral supplementation have been reported to enhance body weight, quality of life (QoL), respiratory muscle strength, and 6-min walk distance (6MWD) and potentially improve lung function [9,10]. Despite widespread investigations that emphasize the above-mentioned benefits [11,12], GOLD guidelines do not provide specific recommendations for nutritional interventions in COPD management. Therefore, we aim to review here the recent strategies and achievements in the field of nutritional support of COPD patients.

## 2. Assessment Methods and Relevance of Body Composition in COPD

Several measures can estimate body composition that may be linked to lung function and COPD development [13]. Among them, the most frequently used is BMI (body mass index). Decline in BMI in non-obese and increase in BMI in obese patients are associated with worse mortality outcomes and elevate the risk of exacerbations [14]. It has long been shown that malnutrition in COPD patients (as defined by low BMI or body weight) is associated with functional impairment. It adversely affects skeletal muscles, including the respiratory muscles, leading to decreased diaphragmatic mass and diaphragmatic strength that results in respiratory failure. Consequently, undernutrition is linked to increased dyspnea and greater air trapping [15,16,17]. Moreover, malnourished individuals also typically exhibit more impaired diffusion capacity [18]. These effects extend beyond the respiratory muscles, contributing to decreased endurance and mobility as well [19]. Consequently, malnutrition in COPD is linked to diminished health-related QoL, worse prognosis and outcomes, as well as elevated healthcare use and costs [19,20,21,22]. However, although BMI was extensively studied in relation to COPD in the past, more comprehensive methods that better reflect body composition are available nowadays. Recent studies have emphasized the importance of muscle mass rather than just overall weight gain in COPD patients. An increase in muscle mass, rather than weight, in malnourished COPD patients has been shown to correlate with improved survival and better long-term outcomes [23,24]. Skeletal muscle index (SMI) and fat-free mass index (FFMI) are measures of muscle mass that are also reliable indicators of lung function, COPD severity, and risk of mortality [25,26,27,28]. Additionally, the treatment of COPD, e.g., with corticosteroids, also induces muscle wasting and loss in bone mineral density, emphasizing the necessity of combined exercise and nutritional supplementation to increase muscle mass in COPD patients [29]. Markers of obesity, such as body fat percentage (BFP), waist circumference (WC), waist-to-hip ratio (WHR), and visceral fat area (VFA), are inversely associated with lung function and positively correlate with COPD incidence [28,30,31,32]. On one hand, fat deposition within the airway wall may directly affect lung function; on the other hand, whole-body fat accumulation leads to metabolic and hormonal imbalance that increases systemic inflammation, a key factor in COPD [33,34].

The phenotype of COPD is also linked with changes in body composition. In advanced stages of the disease, a typical chronic bronchitis patient, often called a “blue bloater”, presents as obese. In contrast, a typical emphysema patient, known as a “pink puffer”, typically appears cachectic. Indeed, emphysema patients have significantly lower BMI compared to those with bronchitis, primarily due to reduced lean mass, fat mass, and bone mineral content. Additionally, chronic bronchitis patients have higher fat mass and lower bone mineral content compared to healthy controls [35,36]. This emphasizes the necessity of personalized management for nutritionally imbalanced COPD patients.

For nutritional support, it is recommended that undernourished (underweight, sarcopenic, or cachectic) COPD patients aim for an intake of 35–45 kcal/kg body weight/day and around 1.2 g protein/kg body weight/day, with a focus on essential amino acids [37]. For obese patients (including sarcopenic obese), energy intake should not exceed the recommendations for healthy adults expressed in kcal/kg body weight, but should also focus on protein intake and physical exercise to preserve muscle mass and respiratory muscle function [38]. While carbohydrate intake is usually recommended to make up 50–55% of total energy (5–6 g/kg), recent research suggests that lower carbohydrate consumption could offer benefits by reducing inflammatory markers and blood sugar levels [39,40,41,42]. Moreover, it is widely accepted that, besides their quantity, the quality and composition of different food components are also meaningful determinants of health outcomes. This is of particular importance for low-income populations that tend to concentrate their energy intake on low-cost and limited varieties of foods that may make them vulnerable to chronic diseases [43].

## 3. Impact of Different Dietary Components on COPD Patient Outcomes

**Figure 1:**
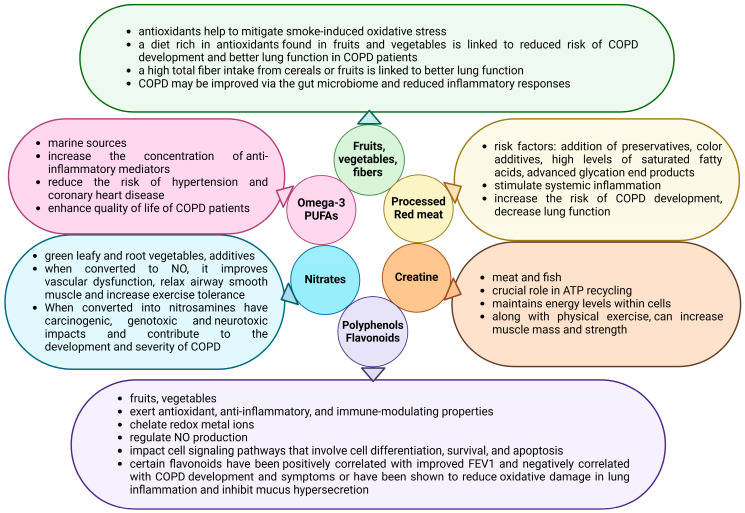
Dietary components and their effects on the welfare of COPD patients. The detailed description can be found in the Section 2 and Section 3.

Various dietary components have been linked to the development and severity of COPD. Main dietary components and their effects on COPD patients are discussed below and summarized in Figure 1.

Research exploring the connection between nutrition and COPD indicates that consuming processed red meat is associated with an increased risk of COPD development, decreased lung function, and a higher rate of COPD-related hospital readmissions [44,45]. However, results are often influenced by bias, and research has shown that not the meat itself but its quality and the additives are responsible for these effects. Identified risk factors include the addition of preservatives, color additives, high levels of saturated fatty acids, and advanced glycation end products. These factors are known to stimulate systemic inflammation or have been associated with reduced lung function in COPD patients [46,47,48]. Harmful effects are more pronounced when antioxidant intake is insufficient in males and smokers [45].

Short-chain polyunsaturated fatty acids, PUFAs, such as linolenic acid (LA, omega-6 PUFA) and α-linolenic acid (ALA, omega-3 PUFA), are primarily found in plant seeds and are considered essential fatty acids. The best-studied long-chain PUFAs, eicosapentaenoic acid (EPA, omega-3 PUFA) and docosahexaenoic acid (DHA, omega-3 PUFA), are mainly found in marine sources. ALA can be converted into EPA and then to DHA, but the conversion is very limited, especially in men [49]. Therefore, consuming EPA and DHA directly from foods and/or dietary supplements is the only practical way to increase levels of these fatty acids in the body. EPA and DHA are known to increase the concentration of anti-inflammatory mediators and decrease the expression of adhesion molecules, which may be advantageous for patients with COPD [50]. Additionally, they confer multiple cardiovascular benefits, including reducing the risk of hypertension and coronary heart disease, known comorbidities of COPD [51]. Current recommendations advocate for a daily intake of omega-3 PUFAs to achieve health benefits, especially considering the low consumption of these products in many populations [52]. It should be emphasized that omega-3 PUFAs exhibit anticoagulant properties. However, a recent meta-analysis found no evidence to support the concern raised with regard to the application of omega-3 PUFAs and the potentially increased risk for the occurrence of adverse bleeding manifestations [53]. Recent studies on the effects of PUFAs on COPD suggest that higher n-6 PUFA levels and lower n-3 PUFA levels may be associated with elevated pro-inflammatory markers, decreased muscle mass, diminished QoL, and an increased risk of COPD [54,55,56,57,58,59,60].

Creatine plays a crucial role in recycling ATP, particularly in muscle and brain tissues, and acts as a buffer, helping to maintain energy levels within cells. It is abundantly found in meats and fish and is often consumed as a dietary supplement [61]. It is well-established that creatine supplementation, along with physical exercise, can increase muscle mass and strength in healthy individuals [62,63]. Consequently, several randomized controlled trials have explored the effects of creatine supplementation combined with physical exercise in patients with COPD. They show that there might be a beneficial effect of creatine either alone or in combination with Coenzyme Q10 (CoQ10) on muscle mass. As mentioned above, increased muscle mass is a strong independent predictor of all-cause mortality. However, exercise performance improved only in the study that added CoQ10 to creatine supplementation [64,65,66,67]. Nevertheless, most studies on this topic showed significant limitations [68]. It is also important to note that no safety issues can be detected when consuming creatine long-term [62,63].

Diets rich in fruits and vegetables are suggested to protect against COPD due to their antioxidant properties. Studies based on self-reported questionnaires have shown that individuals who consume more fruits and vegetables are at a reduced risk of developing COPD [69,70]. These protective effects are suggested to be more pronounced in smokers, which might be explained by the fact that antioxidants help to mitigate smoke-induced oxidative stress [45,71,72]. A three-year prospective study found that increasing the intake of fresh fruits and vegetables led to improved lung function in COPD patients across all GOLD stages [73].

Consuming the recommended amount (~14 g/1000 kcal) of dietary fiber, compared to reduced amounts, is linked to better lung function and a decreased risk of developing COPD, especially in current and ex-smokers [74,75,76,77,78]. The protective effect of fiber intake was observed with the consumption of total dietary fibers, cereal fibers, or fruit fibers. Interestingly, no significant effect of increasing vegetable fiber intake was found [77,79]. This might be due to the reduced production of short-chain fatty acids (SCFAs) by the gut microbiome from vegetables high in cellulose or insoluble fibers [80,81]. It is also important to mention that dietary fibers can affect mineral and glucose absorption, which also influences COPD risk (see below) [82,83].

Nitrates are found in the diet mainly in green leafy or root vegetables and are commonly used as additives for curing meats. After consumption, nitrate (NO_3_^−^) is converted to nitrite (NO_2_^−^) and then to nitric oxide (NO) [84]. NO is a powerful vasodilator and contributes to the regulation of blood flow, mitochondrial biogenesis, mitochondrial respiration, glucose uptake, and muscle relaxation [85,86]. In line with that, beetroot juice supplementation has been found to increase plasma NO_3_^−^ levels in COPD patients and, moreover, to improve both vascular dysfunction and exercise tolerance [87,88,89,90]. On the other hand, NO_2_^−^ can react with proteins and be converted into nitrosamines, particularly during high-heat cooking methods like frying or baking. The formation of nitrosamines highly depends on the NO_2_^−^ and protein content of the food [91]. Nitrosamines are also highly prevalent in cigarette smoke. They can lead to detrimental effects, such as carcinogenic, genotoxic, and neurotoxic impacts, and can contribute to the development and severity of COPD [92,93,94,95]. It has been suggested that the presence of antioxidants may inhibit the generation of nitrosamines, and thus the potential harmful effects of NO_2_^−^ and NO_3_^−^ may depend on the levels of antioxidants present in the food [96,97,98]. Moreover, antioxidants can inhibit nitrosamine-induced oxidative stress, cytotoxicity, and genotoxicity [99,100,101].

Polyphenols are a diverse group of plant-derived compounds. Due to their significant structural diversity and differences in their bioavailability, polyphenols influence multiple signaling pathways that may play a significant role in COPD management [102,103,104,105,106]. Polyphenols exert antioxidant, anti-inflammatory, and immune-modulating properties [107,108,109,110,111]. They chelate redox metal ions or act as mild prooxidants, indirectly enhancing the antioxidant defense system by stimulating the production of antioxidants like glutathione [109,112,113]. These effects are beneficial because it has been reported that sustained inflammation in COPD worsens the condition of patients and increases the risk of lung cancer development [114,115]. Polyphenols also impact key cell signaling pathways, such as NF-κB, Nrf2, and PI3K, that regulate gene expression and processes such as differentiation, cell survival, and apoptosis. In addition, they also regulate carbohydrate metabolism via improving glucose tolerance, insulin secretion, and glucose uptake [116,117]. By regulating NO production, they can relax both blood vessels and airway smooth muscle [118,119,120].

Flavonoids belong to the polyphenolic bioactive compounds that are prevalent in a variety of foods such as fruits, vegetables, tea, red wine, chocolate, and coffee. Experimental data suggest that flavonoids may help to prevent the progression of pulmonary emphysema [121,122]. In line with that, certain flavonoids have been positively correlated with improved FEV1 and negatively correlated with COPD symptoms such as chronic cough, shortness of breath, and phlegm production [123]. Flavonoid intake is also inversely related to the prevalence of chronic respiratory diseases and with COPD, however, only in ex- and current smokers [103,124]. Quercetin is among the most potent and extensively investigated flavonoids for COPD. It both reduces TNF-α levels and inhibits elastase release from activated neutrophils, contributing to improved lung elasticity and prevention of emphysema development. However, it does not promote the regeneration of damaged alveoli [125,126]. In addition, quercetin reduces oxidative damage to epithelial cells in lung inflammation [127] and inhibits MUC5AC expression in human airway epithelial cells, suggesting its potential in addressing mucus hypersecretion in COPD [128]. Notably, a six-month supplementation with quercetin significantly decreased pro-inflammatory biomarkers in the bronchoalveolar lavage fluid of patients with COPD [105].

Stilbenes are a class of polyphenolic compounds found in various plants, especially in response to environmental stressors. Resveratrol, the best-known stilbene, was shown to reduce cigarette smoke induced IL-8 production in alveolar macrophages in vitro [104]. Six-week supplementation with resveratrol demonstrated improvements in arterial stiffness, myocardial perfusion, and walking distance in patients with COPD [106]. However, as other polyphenols, resveratrol also has pro-oxidizing properties, and the advantageous influence of resveratrol and pomegranate juice in COPD has also been debated [129,130,131].

The spread of chronic inflammatory diseases in the last centuries led researchers to develop different explanations for that phenomenon. The epithelial barrier hypothesis emphasizes the importance of barrier dysfunction in different organs in the development of chronic inflammatory diseases. The connection is based on shared environmental factors, including smoking, which, on one hand, disrupt the gut microbiome and, on the other hand, damage the lung and gut epithelial integrity. This ultimately contributes to systemic chronic inflammatory processes exacerbating pulmonary inflammation and airway remodeling [132,133,134]. Moreover, it has been suggested that besides systemic actions, disrupted balance in a specific organ, such as the gut, can influence inflammatory state and disease development in another organ, such as the lungs. The gut-lung axis refers to the bidirectional communication between the gut and the lungs, mediated by immune interactions, metabolic byproducts, microbial communities, and the systemic circulation. Emerging evidence emphasizes the role of the gut-lung axis in COPD [135,136]. This is supported by the fact that COPD patients have a higher prevalence of gastrointestinal disorders, including atrophic gastritis and impaired nutrient absorption. Moreover, epidemiologic studies show a significantly increased risk of Crohn’s disease and ulcerative colitis [137,138,139].

Given the interplay between inflammatory processes in the gut and the lungs, microbiome-targeted interventions may offer useful therapeutic strategies for COPD management. Supplementation with probiotics, particularly Lactobacillus and Bifidobacterium strains, has shown immunomodulatory effects and enhanced epithelial barrier integrity [140,141,142]. Probiotic- or metabolite-based dietary interventions that result in elevated SCFA levels in the gut have been linked to improved lung function and suppression of both oxidative stress and inflammation [143,144]. Fecal microbiota transplantation (FMT) is an emerging but experimental approach that might restore gut–lung immune homeostasis in COPD [145,146]. However, while these approaches are promising, long-term clinical trials are needed to assess their efficacy, safety, and sustainability in altering disease progression.

## 4. Effects of Vitamins on the Quality of Life of Patients with COPD

### 4.1. Vitamin A

Vitamin A is a fat-soluble nutrient, primarily found in the diet as retinol (in animal products either as free retinol or attached to a fatty acid as a retinyl ester) and as provitamin A (certain carotenoids in plant-based foods) that contain β-ionone rings that allow them to be converted into retinol in animals possessing the necessary enzymes. Free or all-trans-retinol is esterified to retinyl esters and stored in the liver, mostly in the stellate cells. In the tissues, both all-trans-retinol and carotenoids containing β-ionone rings can be oxidized to all-trans-retinal. This retinal is then converted to all-trans-retinoic acid, the active metabolite of vitamin A [147].

Vitamin A is vital for several essential functions in the body. By binding to nuclear receptors, it regulates gene expression, thus playing an important role in cell growth, differentiation, apoptosis, metabolism, cellular repair, and tissue regeneration [148,149,150,151,152,153,154]. Vitamin A is essential for maintaining a healthy immune system. It supports the production and function of white blood cells and helps to maintain the integrity of mucosal barriers in the skin and the lining of the respiratory and gastrointestinal tracts [155,156]. β-carotene and other carotenoids have direct antioxidant properties, however, the rapid conversion of β-carotene to retinol reduces its direct antioxidant activity (retinol lacks the conjugated double-bond structure), making the role of vitamin A as an antioxidant more indirect [157].

With regard to lung health, retinoic acid is known to play a role in alveolar development, pulmonary cell differentiation, maintenance of epithelial tissue integrity, tissue repair, and immune function, which are important factors in COPD [147,158,159]. Additionally, retinoic acid helps maintain collagen and elastin levels in tissues, organize extracellular matrix and basement membrane structures, and regulate TGF-β levels, thereby inhibiting inflammatory processes [160,161,162,163,164,165,166]. Cigarette smoke exposure induces vitamin A depletion in animal models that is associated with emphysema development [167,168]. In line with that, evidence from in vitro studies and animal models has suggested a possible link between vitamin A levels and COPD development [169,170,171].

Several large trials, such as the Carotene and Retinol Efficacy Trial (CARET), results from the National Health and Nutritional Examination Survey (NHAES), the Third National Health and Nutrition Examination Survey (NHANES III), and the Coronary Artery Risk Development in Young Adults (CARDIA) have demonstrated that serum carotenoid and retinol levels or dietary intakes are positively associated with lung function, while negatively associated with the incidence of COPD [172,173,174,175,176,177,178,179]. However, more complex or conflicting results have also been published [180,181].

Despite the worldwide decrease from 1990 to 2019 in the age-standardized incidence and DALY rates of vitamin A deficiency (VAD), VAD is still a significant public health issue, particularly in low- and middle-income countries, affecting mainly children younger than 5 years of age and men more often than women [182,183]. Therefore, VAD may contribute to the development or progression of COPD, making it essential to ensure adequate vitamin A levels in patients with COPD. According to the Institute of Medicine, the recommended dietary allowance (RDA) for vitamin A is 900 μg and 700 μg for males and females above 70 years, respectively [184]. However, defining the appropriate type and dose of supplementation can be challenging for COPD patients.

**Figure 2:**
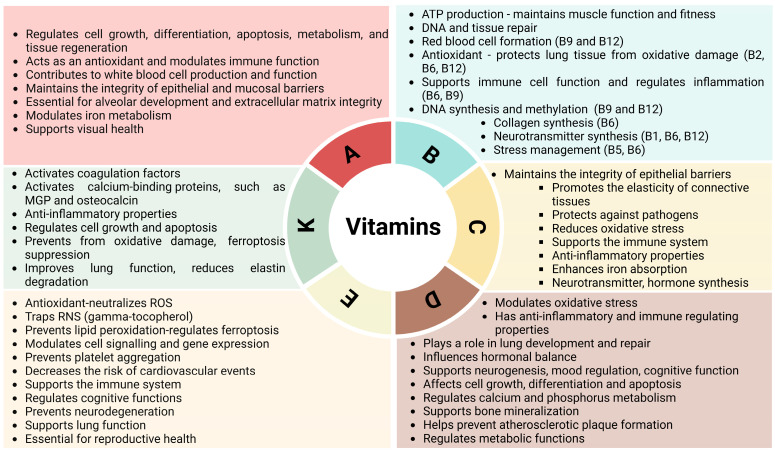
Vitamins and their effects on the welfare of COPD patients. The detailed description can be found in the Section 4.

On one hand, nutritional retinoids have far better utilization rates than carotenoids [185,186,187,188], making it easier to meet vitamin A requirements through retinoid sources. On the other hand, retinol is a relatively unstable compound, and its levels in lung tissue do not correlate directly with those measured in serum. [189]. Moreover, excess retinoid supplementation can result in vitamin A toxicity (a risk usually not associated with carotenoids due to their low and regulated conversion to vitamin A). It has been known for several decades that high levels of retinoids may reduce bone mineral density and might even increase mortality [190,191,192,193]. These effects may be attributed to retinoids functioning as a molecular antagonist to vitamin D [194,195,196,197,198,199,200]. Therefore, it is strongly recommended that vitamin A supplementation always be accompanied by adequate vitamin D levels. Moreover, it has been suggested that carotenoids reduce the synthesis of vitamin D via their photoprotective effect; however, this may not have clinical relevance [201,202,203]. Additionally, carotenoids can be oxidized, and, in specific environments, such as those with high oxidative stress (smoking or asbestos exposure), this oxidation can be accelerated. The increased oxidation rate may contribute to oxidative stress rather than providing protective effects [204,205,206]. The effects of vitamin A and other vitamins on the condition of COPD patients are summarized in Figure 2. The chemical structures of different vitamins or vitamin subtypes are shown in Supplementary Figure S1.

### 4.2. B Vitamins

B vitamins are a group of water-soluble vitamins with highly overlapping roles [207,208,209,210,211]. These include the support of energy production by acting as coenzymes in metabolic processes, thereby helping to maintain adequate muscle function and fitness [212,213]. They possess antioxidant properties that protect lung tissues from oxidative damage [214]. Adequate levels of B vitamins support the immune system and help to protect lungs from infections [215,216]. B vitamins are involved in DNA synthesis, methylation, DNA and tissue repair [210]. Additionally, they also regulate homocysteine levels, potentially reducing the risk of complications, such as atherothrombotic events [211]. B vitamins, particularly vitamins B2 (riboflavin), B3 (niacin), and B6 (pyridoxine), are involved in amino acid metabolism and cellular functions that support collagen synthesis [217,218]. B vitamins play important roles in red blood cell formation, ensuring proper oxygen delivery, which is crucial for COPD patients [208]. In addition, B vitamins are involved in the synthesis of neurotransmitters, essential for brain function and cognitive health [207]. Given that stress is frequently reported by smokers as a significant trigger for high cigarette cravings, B vitamins may aid in stress management and support smoking cessation efforts by supporting neurotransmitter function and regulating mood. B12 vitamin supplementation was shown to improve cognitive impairment following nicotine withdrawal in rats [219]; however, that should be verified in humans.

Although B vitamin deficiencies are generally considered rare, it is important to mention that subclinical deficiencies are difficult to measure and lack a clearly defined cutoff level [220,221]. However, they can still impact health status and QoL. In particular, the recommended intake of vitamin B1 (thiamin) may be insufficient for a significant portion of the Western population. Extensive research suggests that thiamine deficiency is more prevalent than commonly acknowledged and often remains under-recognized. Reported incidence rates vary widely depending on the study population [222,223,224]. Furthermore, providing high doses of thiamine to individuals considered thiamine-sufficient or marginally deficient leads to significant improvements in QoL, highlighting the challenges in accurately defining thiamine sufficiency [225,226]. Similarly, vitamin B12 (cobalamin) subclinical deficiency is present in an estimated 2% to 26% of the population, varying based on the definition and the measuring method applied [220,227]. It is also important to note that the elderly have significantly lower levels of different B vitamins, even in the case of the recommended dietary intake or no signs of comorbidities [228,229,230]. Moreover, both the intake and the serum levels of different B vitamins are reduced significantly in smokers compared to non-smokers [231,232,233,234].

In line with the above, it has been shown that the consumption of B vitamins (specifically folate, thiamin, niacin, pyridoxine, and cobalamin) of COPD patients is lower than that of control subjects and does not meet the dietary recommendations [235,236,237]. Moreover, intakes of different types of B vitamins (thiamin, riboflavin, niacin, folate) are associated with better lung function and reduced risk or decreased severity of airway impairment, specifically in male and current smoker COPD patients [235,237,238,239,240,241,242]. Furthermore, COPD patients with lower vitamin B6 intake have a higher risk of frailty [243]. Additionally, while patients with stable COPD do not have significantly lower vitamin B12 levels compared to controls [244,245], those with an acute exacerbation commonly have vitamin B12 deficiency [246]. Consequently, supplementation with vitamin B12 leads to discrete positive effects on exercise tolerance in those with more advanced COPD [247].

Despite limited and low-quality data, often characterized by small sample sizes, there is evidence suggesting that B vitamin deficiency in individuals with COPD, particularly among those who are undernourished or have comorbidities, may exacerbate symptoms or contribute to broader health complications. Therefore, ensuring adequate intake of B vitamins is crucial for this population.

### 4.3. Vitamin C

The absorption of this water-soluble vitamin occurs through both active transport and facilitated diffusion mechanisms. Sodium-dependent vitamin C transporters (SVCT1 and SVCT2) play critical roles in this process, with SVCT1 being responsible for intestinal uptake and renal reabsorption, while SVCT2 facilitates the cellular uptake of ascorbic acid. Its absorption is more efficient at lower doses due to transporter saturation. Plasma vitamin C levels are tightly regulated, and excess amounts are excreted via the urine. In the body, ascorbic acid can be oxidized to dehydroascorbic acid (DHA) and subsequently to diketogulonic acid (DKG). While DHA can be reduced back to ascorbic acid with the aid of glutathione and other reducing agents, DKG, being a more stable and less reactive compound, is primarily excreted [248,249].

Vitamin C has been shown to play several roles in the body as well as in the context of COPD. The lungs have a relatively high concentration of ascorbic acid in both intracellular and extracellular compartments in order to protect against inhaled pathogens and pollutants and the constant challenge caused by high oxygen levels [250]. The beneficial effects of vitamin C rely on two main mechanisms. Firstly, it is a potent antioxidant. There is direct evidence showing that the levels of reactive oxygen species (ROS) are elevated in patients with COPD [251,252,253]. Ascorbic acid can both directly (by reaction with aqueous peroxyl radicals) and indirectly (by restoring other antioxidants, such as thioredoxin, glutathione, coenzyme Q, and α-tocopherol) help to neutralize free radicals, thereby reducing oxidative stress and potentially mitigating damage to lung tissues [254,255,256,257]. The indirect antioxidant action of vitamin C is possibly mediated via induction of transcriptional factors such as Nrf2 (nuclear factor erythroid 2-related factor 2) and AP-1 (activator protein 1) that modulate the expression of other antioxidants [258,259,260].

The other main mechanism is its anti-inflammatory function. Vitamin C is crucial for the proper functioning of the immune system [261,262]. It supports various immune functions by enhancing the production and function of leukocytes, particularly lymphocytes, and promotes differentiation of monocytes into macrophages [263,264]. It regulates the expression of pro-inflammatory cytokines, such as IFN-γ, IL-6, and TNF-α [265]. The impact of ascorbate on Hypoxia Inducible Factors (HIFs) is critical for the function of immune cells, influencing inflammation and cancer [266,267]. In line with that, data demonstrate that vitamin C has anti-inflammatory properties [268,269,270] that may help to reduce COPD-related inflammation.

Additionally, vitamin C helps to maintain the integrity of epithelial barriers, which are the first line of defense against pathogens and pollutants. It protects lipids from peroxidation, thereby maintaining membrane integrity, which is crucial for lung epithelial cells continuously exposed to oxidative stress [271]. Ascorbic acid also acts as a cofactor for prolyl and lysyl hydroxylase enzymes and is essential for the formation of the triple helix structure of collagen; therefore, it is vital for maintaining the integrity and elasticity of connective tissues, including those in the airways [272,273,274,275].

Both carnitine and catecholamine metabolism are regulated by ascorbic acid, which is not only important for brain function and mood regulation but plays an important role in COPD development as well [276,277,278,279,280,281]. Carnitine is essential for the transport of fatty acids into the mitochondria for energy production, a process that is particularly important in COPD patients to support muscle function and reduce fatigue commonly associated with the disease [282]. Catecholamines, e.g., adrenaline and noradrenaline, are involved in the regulation of airway tone by stimulating beta-adrenergic receptors that result in bronchodilation and improved airflow in the lungs. In addition, catecholamines also modulate inflammatory and immune responses [283]. Moreover, vitamin C has been suggested to have mucolytic properties [284]; however, this is still debated [285].

Vitamin C plays a role in synthesizing and regulating certain hormones, including those involved in stress response and metabolism [286,287,288,289]. Additionally, ascorbic acid converts ferric iron (Fe^3^⁺) to the more absorbable ferrous form (Fe^2^⁺) and forms a soluble complex with ferrous iron at the acidic pH of the stomach, thereby significantly increasing its bioavailability [290]. This enhanced iron absorption may have implications for individuals with COPD.

Vitamin C hypovitaminosis and deficiency are common in low- and middle-income countries and present in high-income countries among specific populations [291]. Both smokers and passive smokers have lower plasma vitamin C levels compared to non-smokers, which can be explained by lower intake, increased oxidative stress, and a higher metabolic turnover of ascorbic acid in smokers [292,293,294,295]. Moreover, patients with chronic respiratory diseases have lower plasma vitamin C levels than healthy matched controls, that is accompanied by higher oxidative stress and inflammatory marker profile [296,297].

In line with that, several studies have suggested that higher serum levels [174,181,298,299,300,301] and higher dietary intake of vitamin C or intake of a combination of different antioxidants including vitamin C are associated with better lung function, reduced risk of COPD or COPD exacerbations [180,240,298,302,303,304,305,306]. However, some studies were not able to show a significant effect of dietary vitamin C consumption on lung function [307,308,309].

Evidence from well-designed trials examining the effects of vitamin C supplementation on COPD is limited and present small sample size. Some studies have been criticized for poor quality and have only partially addressed the impact of ascorbic acid on modifying COPD risk, rather focusing on its impact on exercise tolerance. Most studies suggest that vitamin C supplementation may help protect against oxidative stress and reduce the frequency of exacerbations, although it does not appear to significantly affect lung function [310,311,312,313,314,315]. A recent meta-analysis suggested that vitamin C supplementation as a potent antioxidant may improve lung function in COPD patients with an intake of more than 400 mg/day [316].

The RDA for vitamin C has not changed since 2000, with 90 mg/day for healthy males and 75 mg/day for females. The estimated requirement for smokers ranges between 140 and 200 mg/day to achieve serum ascorbate concentrations comparable to those of non-smokers [294,317]. However, in chronic conditions such as COPD, much higher intake should be necessary. Although the tolerable upper intake limit (UL) for vitamin C in healthy adults is set at 2000 mg, the risk of adverse effects resulting from excess intake appears to be very low. Therefore, while healthy individuals generally do not need to exceed the upper limit, higher doses may be justified in COPD to achieve adequate serum levels [318]. It is important to note that the bioavailability of vitamin C is decreasing after reaching a 200 mg single dose, with about 50% for a single dose of 1000 mg. Thus, continuous supplementation throughout the day may offer greater benefits [319,320]. Moreover, as ascorbate can exhibit pro-oxidant properties under certain conditions, combining vitamin C with other antioxidants might be crucial for effectively reducing oxidative stress in COPD [321,322].

### 4.4. Vitamin D

Vitamin D exists in two different forms: D2 (ergocalciferol), present in plants or fungi, and D3 (cholecalciferol), synthesized in the skin from 7-dehydrocholesterol when exposed to UVB light. Both are converted to the active calcitriol (1,25(OH)_2_D); however, D3 is more effective at maintaining 25(OH)D levels due to its higher affinity for vitamin D binding protein (VDBP) and slower clearance. Moreover, the production of active vitamin D3 is regulated by the conversion of previtamin D3 to a variety of photoproducts in case of UV overexposure [147,323,324,325,326,327].

Calcitriol has been shown to have anti-inflammatory and immune-regulating properties [147,328,329]. Specifically, it reduces the production of pro-inflammatory cytokines, such as TNF-α, IL-1β, and IL-6, and promotes the production of anti-inflammatory cytokines, like IL-10 [330,331]. Calcitriol also influences the activity of various immune cells, including T-cells, macrophages, and dendritic cells [332,333]. By promoting the development of regulatory T-cells, it helps maintain immune tolerance and prevent autoimmune responses [334]. Similarly to vitamin C, calcitriol can inhibit the NF-κB pathway, which reduces the expression of genes linked to inflammation [335]. Moreover, by modulating oxidative stress, calcitriol can also indirectly reduce inflammation [336,337]. Furthermore, calcitriol can suppress the renin-angiotensin system (RAS), which plays a role in inflammation, blood pressure regulation, and neuroprotection [338,339]. Taken together, COPD patients suffer from exaggerated inflammation, increased oxidative stress, and impaired host defense; therefore, vitamin D supplementation may support therapy due to its potent anti-inflammatory, antioxidative, and antimicrobial functions [340].

In addition, calcitriol plays a role in lung development and repair processes [341]. Vitamin D deficiency during lung development can lead to structural abnormalities and impaired lung function [342,343,344,345,346]. Vitamin D deficiency coupled with cigarette smoke exposure results in a COPD-like phenotype in mouse models [347,348]. Moreover, a study using vitamin D receptor knockout mice reported emphysema, reduced lung function, and activation of pathways associated with COPD pathogenesis [349]. In addition, vitamin D influences the synthesis of neurotransmitters, such as serotonin and dopamine, precursors to norepinephrine, and key regulators of airway tone and inflammation [350,351]. Furthermore, by helping to maintain phosphate levels, vitamin D regulates the production of ATP, and its deficiency has been linked to muscle weakness, suggesting a role in respiratory muscle function [352,353].

Calcitriol enhances the absorption of calcium (Ca) and phosphorus by increasing the expression of Ca-binding proteins and sodium-phosphate cotransporters in the small intestine. Additionally, it modulates Ca reabsorption in the renal tubules. Thereby, it not only regulates various metabolic functions, but when accompanied by adequate vitamin K2 and magnesium (Mg) levels, it also aids the mineralization of bone matrix [354,355].

Vitamin D also plays important roles in cardiovascular health, which is often compromised in COPD patients. Besides regulating blood pressure, it also prevents atherosclerotic plaque formation via modulating matrix Gla-protein (MGP) and maintaining Ca balance. Deficiency in vitamin D has been linked to impaired cardiac function [356,357,358,359,360].

Moreover, vitamin D influences cell growth, differentiation, and apoptosis, thus playing a key role in cancer prevention. It regulates the expression of growth factors and modulates hypoxia, which drives key oncogenic and angiogenic pathways. It also promotes pro-apoptotic genes (Bax, Caspases, Fas, p21) while inhibiting anti-apoptotic genes (Bcl-2, Mcl-1, Survivin, Bcl-xL), and can trigger the mitochondrial apoptosis pathway [361,362,363,364,365,366,367]. 

Vitamin D also regulates multiple enzymes involved in the production of steroid hormones [368,369] and plays a role in insulin secretion and sensitivity via modulating the function of pancreatic beta cells [370,371]. Moreover, parathyroid hormone and vitamin D have a reciprocal regulatory relationship [372].

Vitamin D deficiency is prevalent throughout the world. In the past years, numerous epidemiologic analyses have associated vitamin D insufficiency with adverse health conditions [373]. For a long time, vitamin D deficiency has been regarded as a consequence and not a cause of COPD because of the sedentary lifestyle accompanying the disease. However, smokers have lower circulating vitamin D levels than non-smokers [374]. Moreover, vitamin D insufficiency increases the susceptibility to respiratory infections, which are common triggers for COPD development and exacerbations [375,376].

Several studies have investigated the relationship between vitamin D status and COPD. The majority have suggested that low serum vitamin D levels are associated with reduced FEV1, FVC, an increased risk of developing COPD, severity, exacerbations, or mortality of COPD [377,378,379]. The association is stronger in men and current smokers [377,378]. Low vitamin D level has been linked to the development of emphysema, airway wall thickening, chronic bronchitis, and pulmonary fibrosis [380,381,382,383]. Moreover, a causal relationship between ethnicity or genetically regulated 25(OH)D concentrations and COPD risk has also been detected. Certain genetic alterations of the VDBP, such as the rs4588 and the rs7041 polymorphisms, are associated with changes in circulating 25(OH)D concentrations and with the risk of COPD [384,385,386,387,388,389].

In line with that, vitamin D supplementation in patients with stable COPD improves lung function (FEV1, FEV1/FVC) and 6MWD, while reducing sputum volume, COPD assessment test (CAT) score, and the number of acute exacerbation episodes [390]. However, randomized controlled trials on COPD patients show that vitamin D supplementation may reduce the rate of exacerbations in those with baseline 25-hydroxyvitamin D levels < 25 nmol/L but not in patients with higher levels [391,392]. Based on the above results, the GOLD 2024 report recommends that all patients hospitalized for exacerbations should be tested for severe vitamin D deficiency (<10 ng/mL or <25 nM), followed by supplementation if required [393]. However, a recent meta-analysis found negligible effects of vitamin D supplementation on both lung function and decreasing exacerbations [394]. It is important to mention that studies evaluating the effect of vitamin D usually do not address the levels of Mg, zinc (Zn), boron, vitamin K2, Ca, phosphorus, or parathyroid hormone, all of which are important regulators of vitamin D metabolism and activity [395,396,397].

Studies examining the relationship between COPD and vitamin D levels primarily assess whether blood vitamin D levels exceed 25 nmol/L. The Nutrition Examination Survey (2005–2006) determined that the prevalence of vitamin D deficiency was 41.6% in the US (deficiency in this case was defined as 50 nmol/mL serum 25(OH)D) [398]. Research on blood pressure, bone and dental health, incidence of cancer, all-cause mortality, and lower extremity strength indicates that desirable 25(OH)D levels are rather between 90 and 120 nmol/L (36–48 ng/mL) [399]. In a study of Vieth et al., healthy men and women reached desirable blood vitamin D levels when taking 100 μg (4000 IU) vitamin D3/day for 2–5 months [400]. As mentioned above, for smokers or those with a chronic condition, even higher intake may be necessary to reach adequate blood levels. In spite of that, according to the National Institutes of Health, the RDA for elderly is 800 IU with an upper intake UL of 4000 IU. Moreover, some medications commonly used in the management of COPD and its comorbidities (steroids, statins, and thiazide diuretics) impair vitamin D metabolism; therefore, altered vitamin D doses should be considered when taking these medications [401].

However, ensuring vitamin D levels in COPD patients is challenging due to the lack of separate assessments for D3 and D2 forms, the debate over the clinical utility of free vitamin D measurements, the dynamic regulation of calcitriol, the influence of inflammation on 25(OH)D levels, and various external factors such as sun exposure, hormonal status, and assay differences. In line with the mainstream scientific opinion, measurement of serum 25(OH)D provides the best estimate of the vitamin D status [402,403,404,405,406,407,408].

### 4.5. Vitamin E

The vitamin E family consists of four tocopherols and four tocotrienols (each with α-, β-, γ-, and δ members) that are distinguished by the number and arrangement of methyl groups on their chromanol ring. The primary natural sources of tocopherols and tocotrienols are the fatty parts of nuts and oil seeds, oils, fish, egg yolk, and certain vegetables [409]. The liver is key in controlling vitamin E levels by secreting alpha-tocopherol into the bloodstream far more efficiently than other vitamin E isoforms.

The major role of vitamin E is antioxidant defense by neutralizing ROS [410,411,412]. Moreover, compared to other vitamin E isoforms, gamma-tocopherol has a uniquely strong ability to trap reactive nitrogen species (RNS) as well [413,414]. By preventing lipid peroxidation, vitamin E may play important roles in the regulation of ferroptosis, which is particularly important in the development of COPD in response to inhaled heavy metals from cigarette smoke or air pollution. However, direct evidence of the impact of vitamin E on ferroptosis specifically is limited [415,416,417,418].

Different vitamin E isoforms support the immune system through various mechanisms. They regulate T-cell functions [419] and inhibit inflammatory signaling pathways (NF-κB, STAT3, PGE2-COX2 axis), thereby reducing the production of pro-inflammatory cytokines such as TNF-α, IL-1β, and IL-6 [420,421,422,423]. By inhibiting PKC, vitamin E isoforms not only modulate membrane structure and immune responses but also regulate cell fate and cognitive functions [424]. In line with that, epidemiological data suggest that vitamin E is a negative risk factor for certain types of cancer [425,426].

Vitamin E plays crucial roles in cardiovascular health by preventing lipid, particularly LDL cholesterol oxidation, blocking vascular inflammation, inhibiting platelet aggregation, and regulating blood pressure. These effects may help mitigate cardiovascular complications in COPD patients at higher risk for atherosclerosis and related cardiovascular issues [427,428,429,430,431].

Regarding the different chemical forms, although α-tocopherol is considered the dominant form and is the best-studied vitamin E subtype, recent research data indicate that, despite their lower concentrations, other tocopherols, tocopherol metabolites, and tocotrienols play unique and important roles and may provide even broader protection than alpha-tocopherol [432,433,434].

Vitamin E deficiency is usually defined as low serum or tissue α-tocopherol levels. In developed countries, it is regarded as a rare condition that rather reflects abnormalities in dietary fat absorption or metabolism rather than low dietary vitamin E intake. Nonetheless, epidemiological data indicate that low vitamin E intake and insufficiency are common phenomena throughout the world, even in developed countries [435,436,437].

Cigarette smoke induces significant oxidative and nitrative stress. Although plasma levels of both α-tocopherol and γ-tocopherol may not differ significantly between smokers and non-smokers, cigarette smoke leads to α-tocopherol depletion and induces γ-tocopherol nitration [438,439,440,441,442]. Moreover, blood levels of vitamin E are further decreased during exacerbations [439,443].

Numerous studies indicate that higher serum levels—typically referring to α-tocopherol, although not always explicitly defined—and dietary vitamin E intake reduce oxidative stress markers, the risk of wheeze, are positively correlated with improved lung function (FEV1, FVC) and are inversely related to the development of COPD [175,181,307,444,445]. On the other hand, lower serum γ-tocopherol is associated with higher FVC, lower odds of wheeze, and decreased risk of COPD [297,446,447]. However, studies evaluating the effect of α-tocopherol supplementation on lung function have shown mixed results [310,445,446,448,449,450]. Limited research has been conducted on the role of tocotrienols in COPD. However, animal studies demonstrate the ability of γ-tocotrienol to provide antioxidant protection, mitigate inflammation, prevent emphysema development, and preserve lung function in models of COPD [451,452].

Interestingly, those who take vitamin E supplements have significantly higher α- and lower γ- and δ-tocopherol levels than non-users. These findings suggest that supplementing with α-tocopherol reduces the availability of γ- and δ-tocopherol that can be attributed to the dominance of α-tocopherol in most supplements, its preferential absorption and retention by the body, as well as the competitive or metabolic interactions between different tocopherol isoforms [312,446,453]. However, not only oxidative but also nitrative stress plays a significant role in the pathophysiology of COPD [454]. Given that only γ-tocopherol of the vitamin E isoforms has the ability to neutralize RNS, excess supplementation with α-tocopherol may limit the ability of γ-tocopherol to scavenge RNS [455]. It is also important to mention that the RDA for vitamin E is based exclusively on α-tocopherol, suggesting that between 15 and 1000 mg/day is a desirable intake level for adults [456]. Given the complexities surrounding vitamin E isoforms, future high-quality studies are needed to more thoroughly investigate the distinct functions, effects, and efficacy of various vitamin E isoforms.

### 4.6. Vitamin K

There are two main natural forms of vitamin K: K1 (phylloquinone), found in plant-derived foods such as leafy green vegetables, and K2 (different types of menaquinones), found in natto, animal products, fermented foods and also produced by certain bacteria in the gut microbiome [457,458]. Vitamin K1 is primarily absorbed by the liver, where its main role is to activate coagulation factors, including prothrombin and clotting factors II, VII, IX, and X [459]. In contrast, vitamin K2 not only has higher bioavailability [460,461], but it is also more lipophilic, and due to its longer plasma half-life, it more effectively penetrates extrahepatic tissues.

The main function of vitamin K2 is to activate Ca-binding proteins, such as MGP and osteocalcin, through γ-carboxylation. These proteins are primarily produced in response to vitamin D, with vitamin A also playing a significant regulatory role [356,462]. MGP inhibits the calcification of blood vessels, soft tissues, and elastin, thereby helping to maintain the flexibility of both vessels and lungs, which is essential for proper respiratory function [463,464]. Osteocalcin regulates the function and survival of both osteoblasts and osteoclasts [465,466], which in turn influences the deposition of Ca into the bone matrix [467,468].

Vitamin K2 has been shown to have anti-inflammatory properties by modulating the activity of various inflammatory mediators and pathways, such as interleukins, TNF-α, and NF-κB [469,470,471,472]. Moreover, it is an important regulator of cell growth and apoptosis, which may have implications in cancer prevention [473]. Additionally, both vitamin K1 and K2 help to reduce lipid peroxidation and thus oxidative damage [474,475].

Recent studies have demonstrated that fine particulate matter, heavy metals, and organic compounds in polluted air and cigarette smoke can induce lipid peroxidation, a critical process in ferroptosis. Ferroptosis is driven by metal toxicity, most importantly by iron, and contributes to the pathogenesis of COPD [476,477,478,479,480,481]. It has been shown that vitamin K (both K1 and K2) plays a role in ferroptosis suppression [482]. In line with that, higher vitamin K intake or levels have been linked to lower mortality rates, better lung function, reduced elastin degradation, and decreased risk of emphysema, as indicated by blood levels of biomarkers responsible for crosslinking elastin fibers (desmosine and isodesmosine) [178,179,483,484]. Additionally, COPD is frequently accompanied by multimorbidity, such as osteoporosis, fractures, cardiovascular diseases, chronic kidney disease, and sarcopenia, with excess calcification playing a major role in these conditions. Therefore, while both vitamin K1 and K2 reduce oxidative stress, vitamin K2 provides additional benefits in inhibiting the pathogenesis of both COPD (emphysema) and its comorbidities through an MGP-dependent pathway [485,486].

In addition to a diet low in vitamin K, deficiencies can also result from malabsorption issues, gastrointestinal disorders, prolonged antibiotic use, and interactions with certain medications, particularly anticoagulants. Vitamin K deficiency is usually assessed by prolonged prothrombin time, an indirect measure of phylloquinone, though it is nonspecific and changes only when the deficiency is significant [487]. Meanwhile, serum menaquinone analysis is complex and not routinely used [488]. Vitamin K deficiency is considered a rare condition; however, the prevalence of functional vitamin K2 insufficiency (as measured by plasma desphospho-uncarboxylated MGP levels) is ~30% in a middle-aged population, while being ~50% among elderly and those with hypertension, type 2 diabetes, chronic kidney disease, and cardiovascular disease [184,489].

The adequate intakes (AI) of vitamin K are 120 and 90 μg/day for men and women, respectively. As no adverse effects have been reported when consuming higher amounts, no UL has been established [184]. However, due to their very long half-life, high doses of menaquinone-7 supplementation may interfere with oral anticoagulant treatment [490]. It is important to note that recommendations for vitamin K generally focus on the needs related to blood clotting, which is primarily influenced by phylloquinone. Although menaquinone-7 and other forms of vitamin K2 are becoming more recognized for their role in bone and cardiovascular health, vitamin K2 is not yet considered a primary component in the standard guidelines for vitamin K intake.

It is also important to mention that vitamins A and E, especially at high levels, can influence vitamin K metabolism and action. As mentioned earlier, vitamin D regulates the expression of osteocalcin and MGP, that are subsequently activated by vitamin K and Mg. However, excess intake of vitamin A disrupts this balance, potentially through its antagonistic actions on vitamin D signaling [194,195,196,197,198,199,200]. Moreover, α-tocopherol or α-tocopherol quinone also regulates vitamin K activity [491,492,493,494]. These interactions emphasize the importance of a balanced intake of fat-soluble vitamins to maintain their individual and synergistic functions. 

## 5. Effects of Inorganic Elements on the Prognosis of Patients with COPD

**Figure 3:**
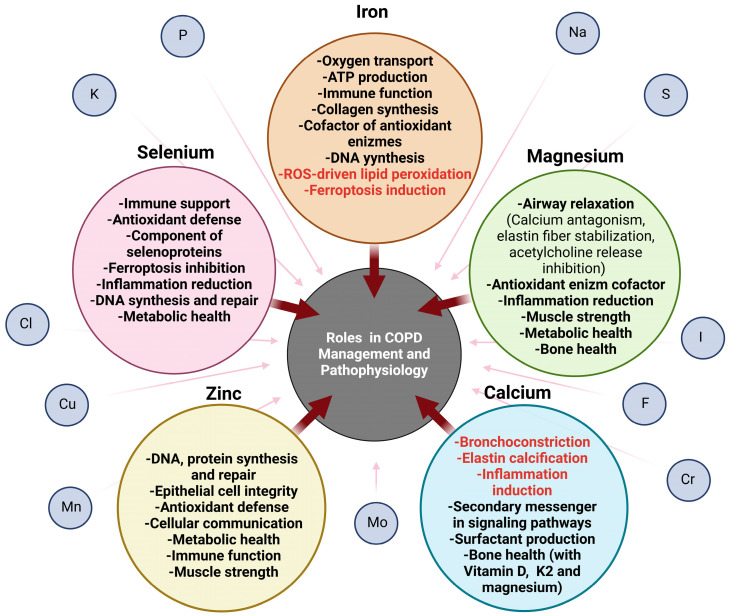
The effects of nutritional inorganic elements on the health of COPD patients. The detailed explanation can be found in the Section 5.

Iron is essential for oxygen transport as being a major component of hemoglobin and myoglobin [495]. It is also involved in ATP production through its role in the electron transport chain [496]. Consequently, decreased levels of iron may lead to fatigue and sarcopenia, which are common conditions in COPD [497]. Iron plays a significant role in immune function [498]. It is a cofactor for certain enzymes responsible for antioxidant actions, such as catalases and peroxidases [499,500], or collagen synthesis, such as prolyl and lysyl hydroxylases [501,502]. Iron is necessary for the synthesis and repair of DNA, supporting cell growth and reproduction [503,504]. The role of iron and other nutritional inorganic elements on the prognosis of COPD patients is summarized in Figure 3.

Heme iron, found in animal sources, is absorbed as an intact molecule with 15–35% efficiency. Non-heme iron is found in plants and requires reduction to the ferrous form for absorption. This process is influenced by a number of dietary factors, with phytates, polyphenols, oxalate, Ca, Zn, and certain proteins having an inhibitory role. In addition, low stomach acid levels, common in conditions like gastroesophageal reflux disease or aging, can also suppress iron absorption. On the other hand, vitamin C and animal-derived foods enhance non-heme iron uptake, resulting in a 2–20% absorption rate [505,506].

Adequate iron supply and homeostasis depend on systemic plasma availability, which is primarily controlled by the hepcidin/ferroportin regulatory system. Elevated iron levels and inflammatory cytokines increase hepcidin production, which inhibits iron absorption and sequesters iron in macrophages and liver cells. This mechanism is a defense strategy by the body to restrict iron availability to pathogens, but it also results in functional iron deficiency during chronic inflammatory conditions like COPD [507,508]. Additionally, COPD can result in hypoxia, which in turn stimulates erythropoiesis to enhance oxygen transport. This increased erythropoiesis necessitates additional iron. Inadequate iron stores to meet this demand may result in anemia [509].

Non-anemic iron deficiency is prevalent among individuals with COPD and has been recognized as a potential risk factor for developing the disease [508]. It is associated with increased hypoxemia and dyspnea, elevated levels of inflammatory markers, and reduced exercise tolerance compared to COPD patients with adequate iron levels [508,510]. In line with that, iron replacement shows a significant decline in serum oxidative stress markers along with an improvement in glutathione levels in patients with stable severe COPD [511]. Moreover, the administration of intravenous iron is associated with improved exercise capacity and QoL [512,513].

On the other hand, excessive iron increases oxidative stress, as it can catalyze the formation of ROS through the Fenton reaction [514]. The airways are exposed to exogenous inhaled iron sources on a continuous basis, as iron is commonly detected in atmospheric particulate matter [515]. Furthermore, habitual cigarette smoking significantly elevates lung exposure to iron. While a small proportion of inhaled iron may enter the systemic circulation, it primarily accumulates in the lung tissue, generating adverse effects. In line with that, smokers exhibit higher levels of total non-heme iron and ferritin in bronchoalveolar lavage (BAL) fluid and alveolar macrophages, as well as increased serum ferritin concentrations, compared to non-smokers [516,517]. Smokers also have elevated levels of redox-active iron in exhaled breath condensate. Interestingly, in patients with COPD, these levels are reduced, possibly due to the sequestration of iron by alveolar macrophages as a protective mechanism against oxidative stress [518,519]. Therefore, for iron-deficient COPD patients, iron supplementation may be necessary but should be managed carefully to avoid potential complications related to excess iron.

Mg is an essential mineral that plays a crucial role in numerous physiological functions. It acts as a natural Ca antagonist, reducing Ca influx into muscle cells, which helps to prevent excessive airway contraction [520]. Mg supports the stability and integrity of elastin fibers by aiding proper collagen and elastin cross-linking, a process that is also influenced by Ca [521]. By inhibiting the release of acetylcholine, a neurotransmitter that causes bronchoconstriction, Mg further promotes airway relaxation and improves breathing [522]. It is also crucial for energy production, helping to maintain respiratory muscle strength and overall physical performance [523]. By regulating Ca levels, it helps to maintain bone mineral density that is also often diminished in COPD [524]. Mg also modulates inflammatory responses by reducing pro-inflammatory cytokine release, thereby mitigating the negative effects of inflammation on the respiratory system [525]. Moreover, Mg plays a key role in maintaining the integrity and function of immune cells, which is vital given the frequent infections and inflammatory responses associated with COPD [526]. As a cofactor for important antioxidant enzymes, Mg supports antioxidant defenses, protects cellular structures, and maintains metal ion balance [527,528,529,530]. It also regulates blood sugar levels and lipid profiles, crucial for managing comorbid conditions, like diabetes and cardiovascular disease, prevalent in COPD patients [531,532,533]. Mg is involved in the synthesis and repair of DNA and RNA, as well as in the activation of amino acid and protein synthesis [534,535]. Furthermore, it facilitates the transmission of electrical signals in the nervous system [536].

Mg deficiency is defined as blood levels of less than 0.6 mmol/L (1.46 mg/dL). Since serum Mg makes up only 1% of the total Mg pool of the body and does not accurately reflect intracellular Mg levels, levels up to 0.85 mmol/L may indicate chronic latent Mg deficiency. This aligns with the fact that around half of the US population has been shown to consume less than the daily requirement of Mg from food [537,538,539,540]. Even with the conventional definition, Mg deficiency is frequently observed in COPD patients, particularly during exacerbations, and its level is correlated with the frequency of exacerbations and overall QoL [541,542]. The low Mg levels may result from inadequate dietary intake, reduced absorption, and increased excretion, which is often related to medications commonly used in the treatment of COPD and its comorbid conditions (corticosteroids, beta-2 agonists, proton-pump inhibitors, antibiotics, and diuretics) [543,544].

In stable COPD patients, some studies show that Mg-sulfate reduces lung hyperinflation, improves respiratory muscle strength and exercise performance when administered in a 2 times 2 g iv. dose [545,546]. On the other hand, oral and only 300 mg daily Mg-citrate supplementation was unable to substantially influence lung function, physical performance, or QoL. However, it may reduce systemic inflammation [547].

Regarding patients experiencing COPD exacerbations, a meta-analysis found that iv. Mg-sulfate, when compared to placebo, may reduce hospital admissions, shorten the duration of hospital stays, and improve dyspnea scores. However, there were no notable differences in the need for non-invasive ventilation, lung function, oxygen saturation levels, or the occurrence of adverse events. Furthermore, the studies were unable to determine the effects of nebulized Mg-sulfate compared to standard care (ipratropium bromide) or placebo. However, as dose and route of administration were not considered in this study, further research is necessary to determine the optimal supplementation method [548,549,550]. The minimum suggested dose for fixing Mg deficiency (defined as Mg levels below 0.80 mmol/L) is 600 mg/day for one month, then continue with a dose that holds serum values no lower than 0.90 mmol/L [551].

Ca impacts various aspects of COPD pathomechanism and management. Excessive Ca influx into respiratory muscles can lead to increased bronchoconstriction, which exacerbates airflow limitation and breathlessness [552,553]. Elevated intracellular Ca levels can enhance the release of pro-inflammatory cytokines, contributing to the chronic inflammatory characteristic of COPD [554]. Additionally, excess Ca not only leads to elastin calcification in the lungs, resulting in decreased elastic recoil, but also causes vascular damage and elevated blood pressure, which are common comorbidities in COPD [521,555,556].

On the other hand, Ca is essential to both surfactant production and mucus secretion [557,558]. It also acts as a secondary messenger in various signaling pathways, which are important regulators of cellular function and stress responses in COPD [559]. Moreover, although data are inconclusive, when combined with other essential nutrients, such as vitamins D and K, Ca may help preserve bone mineral density that is frequently reduced in COPD patients due to chronic inflammation and the use of corticosteroids [560,561,562,563,564,565].

Ca deficiency is defined by serum Ca levels less than 8.5 mg/dL (2.12 mmol/L). Healthy adults in the developed countries usually have efficient Ca intake, and Ca deficiency is considered a relatively rare condition that is frequently a consequence of vitamin D or Mg imbalance [566,567]. These micronutrients influence Ca absorption, while vitamin K2 regulates Ca deposition into the bones [354,355,467,468,568]. With a disruption in the vitamin D-vitamin K-Mg balance, Ca deposition into the vascular structures and calcification of elastin fibers can cause widespread detrimental consequences [566,567,569]. In line with the above, a recent study indicated that hypercalcemia is associated with an increased risk of COPD incidence and mortality [570]. Ca channel blockers might be prescribed to COPD patients to address associated conditions like hypertension, angina, or pulmonary hypertension. A study investigated whether amlodipine, a Ca channel blocker, influences the risk of exacerbations and overall mortality in COPD patients. The results showed that amlodipine was associated with a reduced risk of death from any cause after one year compared to bendroflumethiazide. However, there was no significant difference in the frequency of exacerbations between the two treatments [571].

Zn is an essential trace element playing critical roles in numerous biological processes. It has antioxidant properties that help to protect lung tissues from oxidative stress and inflammation, both of which are important features in COPD [572]. Zn is important for maintaining an adequate immune system and promoting wound healing [573,574]. In addition, Zn helps maintain the structural integrity of respiratory epithelial cells [575]. It is involved in the synthesis, storage, and release of insulin, regulates blood sugar levels, and plays a role in thyroid hormone metabolism that influences overall metabolic rate and energy production [576,577]. Also, Zn is a cofactor for over 300 enzymes, supporting functions such as DNA and protein synthesis, as well as cell division [578]. Zn is also recognized for its role as a regulatory and signaling element in both intercellular and intracellular communication [579].

Zn levels are often reduced in patients with COPD, especially in severe disease [580,581]. Zn supplementation can enhance muscle strength, reduce exacerbation symptoms, and decrease the length of mechanical ventilation [312,314,580,581]. However, maintaining an appropriate balance is crucial, as both Zn deficiency and excess can result in adverse effects [572].

Selenium (Se) is a vital trace element that plays numerous critical roles in maintaining overall health. It is a component of selenoproteins, including glutathione peroxidases and thioredoxin reductases, which are responsible for neutralizing harmful free radicals and contribute to ferroptosis inhibition [582,583,584]. Se also boosts the immune system by enhancing the proliferation of immune cells and increasing the production of antibodies. Moreover, Se can indirectly lower the levels of inflammatory cytokines and other inflammatory mediators [585]. It is involved in DNA synthesis and repair, helping to maintain genomic stability [586].

Although cigarette smoke contains selenium, both serum Se levels and activities of erythrocyte glutathione peroxidase (GSH-Px), a Se-dependent antioxidant, may be lower in tobacco smokers than in non-smokers [587,588,589,590]. In line with that, some studies suggest that higher Se levels are associated with better lung function, improved QoL, and reduced frequency of exacerbations [174,299,312,314,580,591]. Se might act through regulating PUFA-dependent inflammatory pathways, thereby reducing cadmium toxicity from cigarette smoking [592]. Interestingly, expression of Se responsive genes is also altered in COPD [593]. Unfortunately, no effect on 8-iso-PGF2α, an oxidative stress marker, was observed with oral Se supplementation [594]. However, intravenous administration of Se, manganese, and Zn reduced the period patients spent on mechanical ventilation [580].

There are seven essential macro minerals: chloride, phosphorus, potassium, sodium, sulfur, and (as previously discussed) Ca, Mg. Additionally, there are ten essential trace minerals: chromium, cobalt, copper, fluoride, iodine, manganese, molybdenum, and (as previously discussed) iron, Se, and Zn. These minerals are interconnected with one another. To name just a few examples: a lack of sodium can lead to Mg deficiency, Mg helps protect against potassium loss, while higher Mg and potassium intake helps to prevent calcification. Beyond these direct connections, these minerals often work together to support the function of various enzymatic processes. When considering supplementation, it is important to keep in mind that many people suffer from low intake of the variety of other essential minerals. For this reason, supplementation should be achieved in a way that addresses all the key minerals. Moreover, minerals and heavy metals compete for absorption. As a result, heavy metals deplete and impair the functions of minerals in the body. Given that a variety of heavy metals are present in cigarette smoke and polluted air, the main underlying factors of COPD, one of the best ways to prevent heavy metal toxicity in COPD patients, are smoking cessation and consuming a diet rich in dietary minerals [595,596].

## 6. Conclusions

Despite the growing number of studies on the nutritional prevention and management of COPD over the past decades, many questions remain unanswered, primarily due to challenges in experimental design and methodology. On one hand, using food frequency questionnaires to evaluate nutritional status—often retrospectively—introduces the risk of misreporting dietary intake and miscalculating the macro- and micronutrient content of consumed foods. The complexity of human diets makes it difficult to compare results across groups or studies and find all nutritional factors that are responsible for a given effect. Additionally, the bioavailability and pharmacokinetics of most dietary components vary significantly between individuals, further complicating the evaluation of study outcomes. On the other hand, finding an easy, cheap, and preferably non-invasive measurement method of intracellular nutrient levels also poses significant challenges. As mentioned above, serum levels of vitamins and minerals do not necessarily reflect their levels in the cells. Furthermore, although supplemental studies aim to minimize differences in both the study population and the intake of the nutrient of interest, they rarely adequately account for dietary intake or utilization rate. These studies often focus on a single nutrient, overlooking the presence or absence of other food components necessary for achieving the desired effect of the investigated micronutrient.

Despite current improvements in the pharmacological management of COPD, nutritional intervention of both COPD patients and those at risk should be improved. In the developing countries, besides minimizing environmental exposures to air pollutants, ensuring adequate nutrient intake from intrauterine life throughout adulthood may be essential to support lung development and reduce the risk of COPD. In the developed countries, encouraging smoking cessation is essential, both to reduce lung exposure to toxic agents and also because smoking has well-documented effects on micronutrient levels, leading to both deficiencies and altered metabolism. Considering the complexity of the underlying pathological mechanisms in COPD, the concept of supplying a single compound for nutritional intervention may be far too simple to result in long-term benefits. Attempts have been made to shift the oxidant/antioxidant scale in a desired direction by supplementing antioxidant compounds. However, since antioxidants function as both oxidizing and reducing agents and operate within a complex interrelationship, it is particularly important to combine different antioxidant therapies, such as vitamin C, retinol, various vitamin E isoforms, glutathione, coenzyme Q10, and polyphenols. Moreover, excess intake of specific micronutrients disrupts nutritional balance and may contribute to detrimental health consequences, such as excessive calcification or increased lung cancer risk, especially in certain populations like smokers. As ferroptosis and elastin calcification are key concerns in COPD, ensuring adequate levels of vitamin D, vitamin K2, and Mg may be particularly important for reducing the risk of COPD development and limiting disease severity.

As nutrients derived from dietary sources show the greatest benefits in epidemiological surveys, it is essential to fulfill nutritional needs primarily through a balanced diet. Special attention should be paid to consuming food made from good-quality ingredients and avoiding processed foodstuffs. However, given the decreasing micronutrient content of plants and the elevated micronutrient demand of smokers or chronically ill patients, it is hard to cover micronutrient needs solely from food; therefore, checking for deficiencies and, if necessary, supplementing from the possibly most natural sources is also necessary [597]. It is also important to note that current dietary recommendations for vitamin C, vitamin D, or Mg are relatively low, while others do not reflect recent scientific developments, such as in the case of specific vitamin E and vitamin K isoforms. Therefore, recommendations must be updated for the optimal nutritional management of COPD patients or those being at high-risk populations.

Taken together, COPD is a heterogeneous disease mainly of the respiratory tract with underlying diverse genetic backgrounds and aggravating environmental, lifestyle effects such as industrial dust inhalation and, most frequently, tobacco smoking. Additionally, the increased prevalence of infections further worsens the airway inflammation in COPD patients. Due to the complex pathomechanisms and difficulties to treat COPD, both in the western countries and in the developing world, COPD is among the leading causes of mortality. Because COPD management is still a challenge for the clinicians, alternative interventions such as smoking cessation, lifestyle changes from the sedentary life to the moderate physical activity with special attention to the diet may ameliorate patients’ health. Here, we reviewed the effects of dietary components and supplements on the conditions of COPD. Awareness and deliberate efforts for the consumption of less processed meat and more plant fibers, antioxidants with care of adequate intake of vitamins, and inorganic elements (Fe, Mg, Zn, Se) may help to stabilize COPD and reduce the periods of exacerbations.

## Supplementary Materials

The following supporting information can be downloaded at: https://www.mdpi.com/article/10.3390/nu17071149/s1, Figure S1: The chemical structures of different vitamins or vitamin subtypes.
