# Supplementary material for: Nutritional Support of Chronic Obstructive Pulmonary Disease
Source: Nutrients. 2025 Mar 26;17(7):1149. doi: 10.3390/nu17071149 (PMC11990120; doi:10.3390/nu17071149)
Supplement: Supplementary file 1 [file nutrients-17-01149-s001.zip › nutrients-3487938-supplementary.pdf]

Supplementary Material

Figure S1. Chemical structures of the vitamins dissected in the manuscript.

The presented vitamins have been listed as chemical entities. The exact patterns of drawn structure including architecture, double bonds, chiralities as well as heterocyclic sceletons were drawn by means of Chem Office Ultra 12.0 version with utilization of Chem Draw 12.0.

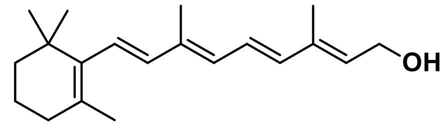

Vitamin A

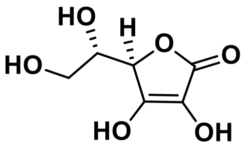

Vitamin C

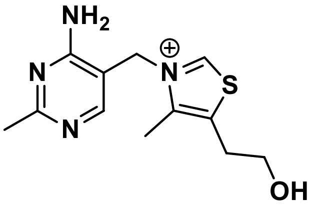

Vitamin B1 (thiamine)

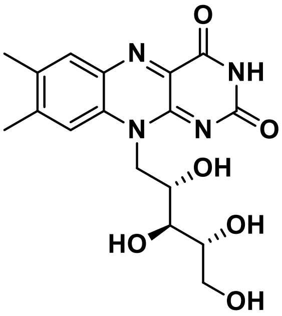

Vitamin B2 (riboflavine)

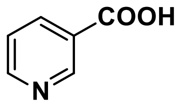

Vitamin B3 (niacin)

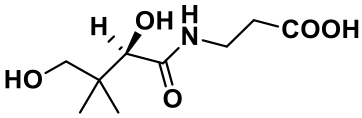

Vitamin B5 (Pantothenic acid)

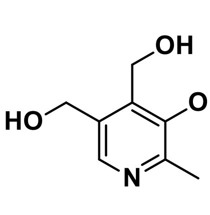

Vitamin B6 (pyridoxine)

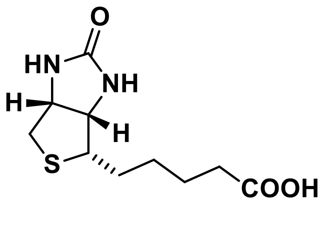

Vitamin B7 (vitamin H)

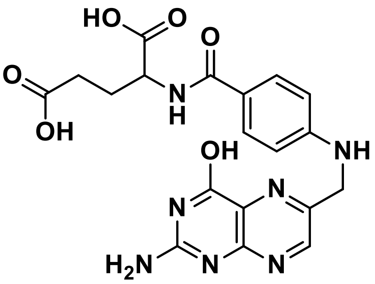

Vitamin B9 (folic acid)

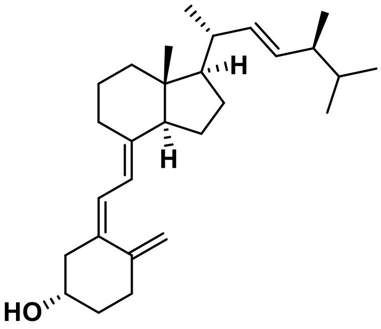

Vitamin D2

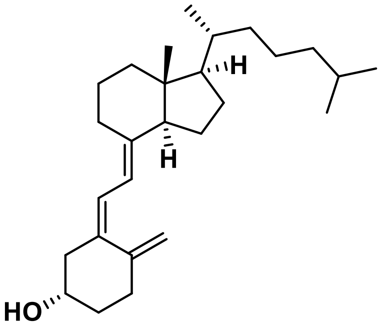

Vitamin D3

Group of Vitamin E homologues

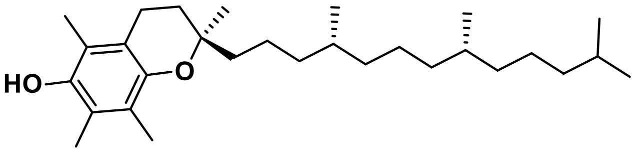

α-tocopherol

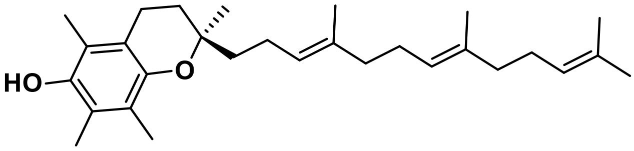

α-tocotrienol

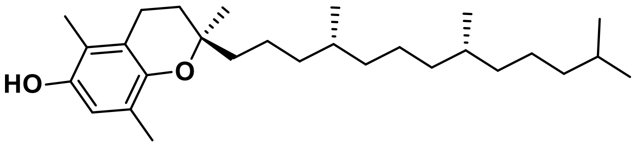

β-tocopherol

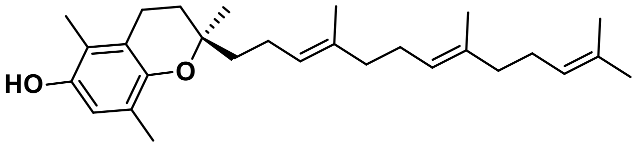

β-tocotrienol

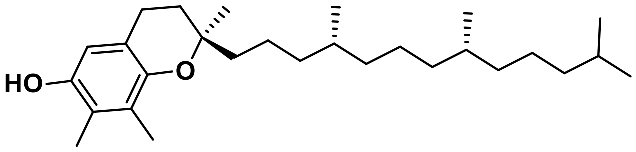

γ-tocopherol

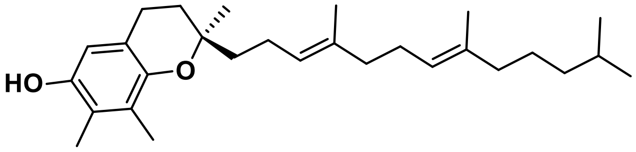

γ-tocotrienol

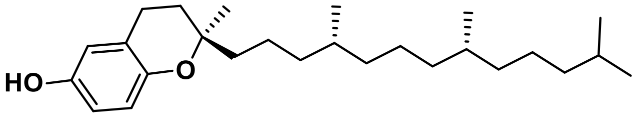

δ-tocopherol

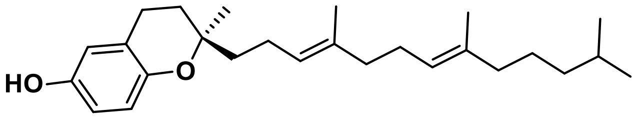

δ-tocotrienol

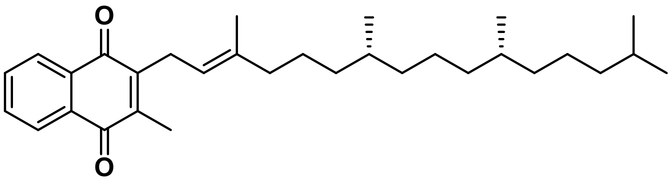

Vitamin K1

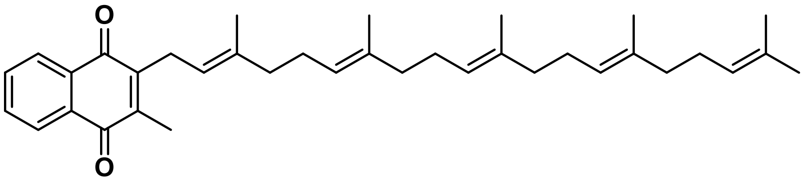

Vitamin K2
